# Internal Introns Promote Backsplicing to Generate Circular RNAs from Spinal Muscular Atrophy Gene

**DOI:** 10.3390/genes13071145

**Published:** 2022-06-25

**Authors:** Diou Luo, Natalia Nikolaevna Singh, Ravindra Narayan Singh

**Affiliations:** Department of Biomedical Sciences, College of Veterinary Medicine, Iowa State University, Ames, IA 50011, USA; diouluo@iastate.edu (D.L.); natalias@iastate.edu (N.N.S.)

**Keywords:** spinal muscular atrophy, SMA, survival motor neuron, SMN, circular RNA, circRNA

## Abstract

Human *survival motor neuron 1* (*SMN1*) codes for SMN, an essential housekeeping protein involved in most aspects of RNA metabolism. Deletions or mutations of *SMN1* lead to spinal muscular atrophy (SMA), a devastating neurodegenerative disease linked to a high rate of infant mortality. *SMN2*, a near identical copy of *SMN1* present in humans, cannot compensate for the loss of *SMN1* due to predominant skipping of *SMN2* exon 7. Restoration of SMN by splicing modulation of *SMN2* exon 7 or gene replacement are currently approved therapies of SMA. Human *SMN* genes produce a vast repertoire of circular RNAs (circRNAs). However, the mechanism of *SMN* circRNA generation has not yet been examined in detail. For example, it remains unknown if forward splicing impacts backsplicing that generates circRNAs containing multiple exons. Here, we employed *SMN* as a model system to examine the impact of intronic sequences on the generation of circRNAs. We performed our experiments in HeLa cells transiently transfected with minigenes expressing three abundantly represented circRNAs containing two or more *SMN* exons. We observed an enhanced rate of circRNA generation when introns joining exons to be incorporated into circRNAs were present as compared to the intronless context. These results underscore the stimulatory effect of forward splicing in the generation of circRNAs containing multiple exons. These findings are consistent with the reported low abundance of *SMN* circRNAs comprised of single exons. We confirmed our findings using inducible HEK 293 cells stably expressing the *SMN* circRNAs. Our results support the role of the exon junction complex in the generation of the exon-only-containing circRNAs. We showed that *SMN* circRNAs were preferentially localized in the cytoplasm. These findings provide new insights regarding our understanding of circRNA generation and open avenues to uncover novel functions of the *SMN* genes.

## 1. Introduction

Circular RNAs (circRNAs) are produced in all eukaryotic cells [1]. Absence of free termini confers high stability to circRNAs [2]. The acknowledged functions of circRNAs include sponging of microRNAs and sequestration of proteins [3,4]. CircRNAs can also code for proteins and regulate transcription [5,6]. Multiple mechanisms, including backsplicing and circularization of lariat RNAs, are associated with circRNA synthesis [7]. Most circRNAs in humans are produced by backsplicing in which the downstream 5′ splice site (5′ss) pairs with the upstream 3′ss [7]. Backsplicing and, hence, circRNA production, are facilitated by inverted Alu repeats present in primate pre-mRNAs due to secondary structure formation [8,9]. Protein factors, including DHX9, QKI and SFPQ, have also been implicated in circRNA synthesis [8,10,11]. DHX9 is a DExD/H-box helicase involved in DNA repair, transcription, pre-mRNA splicing and microRNA processing [12]. QK1 and SFPQ are RNA-binding proteins associated with cell differentiation and neuronal functions, respectively [13,14]. Findings that different types of RNA binding proteins are involved in circRNA generation support the diversity of mechanisms by which circRNAs are produced. However, limited attention has been paid to the role of the upstream and/or downstream splicing events in circRNAs formation.

**Figure 1:**
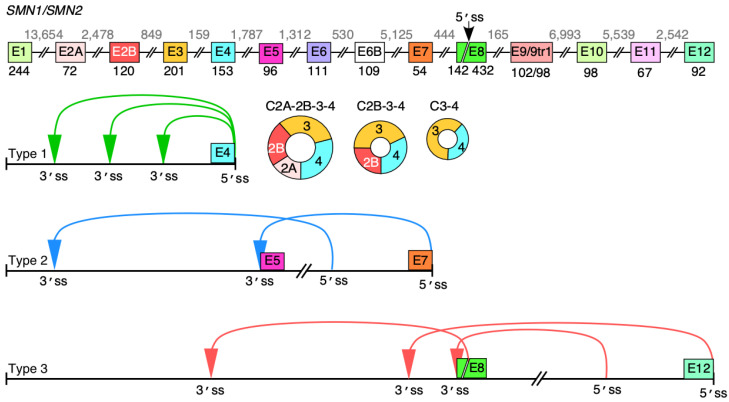
Major backsplicing events in *SMN* genes. Overview of the *SMN* genes and circular RNA they produce. Exons are depicted as colored shapes, while introns are shown as lines/broken lines. Sizes of exons and introns are given below exons and above introns, respectively, in the top panel. A novel 5′ss in exon 8 is marked with a black arrow. All identified circRNAs formed by canonical backsplicing events pertaining to the *SMN* genes are classified into three categories [25]. Type 1 circRNAs are formed by early exons 1 to 4; type 2 circRNAs contain exons 5 to 7 with or without early exons; type 3 circRNAs include the last annotated exon 8 and/or downstream novel exons 9–12. The ranges of exons to form each type of circRNA are marked below the genomic overview. Colored arrows indicate the backsplicing events. The three most abundant type 1 circRNAs are shown as colored circles. The figure was not drawn to scale.

Humans contain two copies of *survival motor neuron* (*SMN*) genes, *SMN1* and *SMN2* [15]. Both genes code for SMN, a multifunction protein essential for the survival of all cell types of the animal kingdom [16]. Mutations or deletions of *SMN1* cause spinal muscular atrophy (SMA), a leading genetic cause of infant mortality [17,18]. *SMN2* cannot compensate for the loss of *SMN1* due to predominant skipping of exon 7. Exon 7 is a coding exon; thus, its skipping results in production of a truncated and unstable protein [19]. SMN levels increase through manipulation of *SMN2* exon 7 splicing using antisense oligonucleotide or a small molecule, which constitute FDA-approved therapies for SMA [20,21]. Gene therapy has also been used to treat SMA [22]. Another avenue to increase SMN levels is through enhanced transcription of the *SMN2* gene, which is almost universally present in SMA patients [23]. With significance to transcription and splicing regulation, the human *SMN* genes harbor a remarkably high number of Alu elements [24]. We and others have recently reported an enormous repertoire of circRNAs generated by the human *SMN* genes [25,26]. Based on their exon composition, we categorized *SMN* circRNAs into four types: Type 1, 2, 3 and 4 [25]. Type 1 circRNAs harbor early exons and use the 5′ss of exon 4 or upstream exons (Figure 1). Type 2 circRNAs harbor both upstream and middle exons. Type 3 circRNAs use the 5′ss located downstream of exon 7 and may incorporate all types of exons, including middle and/or downstream exons (Figure 1). Type 4 *SMN* circRNAs are formed by trans-splicing encompassing one or more exons from another gene.

C2A-2B-3-4, C2B-3-4 and C3-4 are the most abundant circRNAs produced by the *SMN* genes [25] (Figure 1). All three of them contain exons 3 and 4 with or without upstream exons 2A and 2B. Depletion of DHX9, an RNA helicase associated with the unwinding of RNA duplexes formed by inverted Alu repeats, upregulates the C3-4 level at the expense of C2A-2B-3-4 and C2B-3-4 [25]. However, in terms of a complete understanding of the mechanism of *SMN* circRNA production, it remains poorly understood. Here, we employed *SMN* as a model system to examine the impact of intronic sequences on backsplicing during synthesis of circRNAs with multiple exons. Our results obtained using *SMN* circRNA-generating vectors transiently expressed in HeLa cells support that forward splicing of an internal intron joining exons to be included into circRNA favors backsplicing involving a downstream 5′ss. We confirmed our findings employing inducible cell lines stably expressing *SMN* circRNAs. Our results support the role of the exon junction complex (EJC) in the generation of exon-only-containing circRNAs. We showed that *SMN* circRNAs are preferentially localized in the cytoplasm. These findings bring novel insights into our understanding of circRNA generation. The findings are also significant for uncovering novel functions of the *SMN* genes independent of SMN protein functions.

## 2. Materials and Methods

### 2.1. HeLa Cell Culture

HeLa cells (American Type Culture Collection, ATCC) were cultured in Dulbecco’s modified Eagle’s medium (DMEM, Gibco, Grand Island, NY, USA, Cat No. 11965-092), supplied with 10% fetal bovine serum (FBS, Gibco, Cat No. 26140-079). All cell culture media and reagents were purchased from Life Technologies, Carlsbad, CA, USA. Cells were cultured in CO_2_ incubator (Thermo Fisher, Waltham, MA, USA, NAPCO Series 8000WJ) at 37 °C and 5% CO_2_.

### 2.2. Generation of circRNA Overexpression Constructs and Their Linear Counterparts

**Figure 2:**
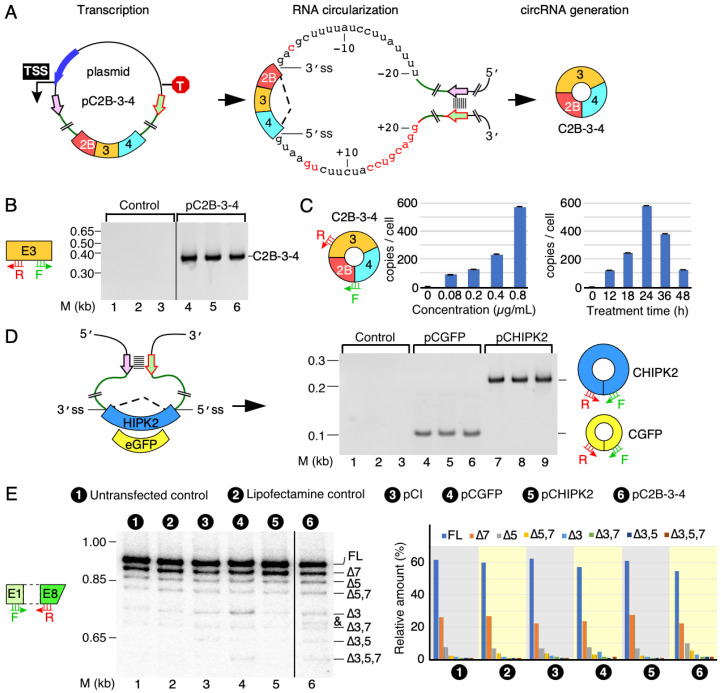
The expression of circRNAs using engineered expression vectors. (**A**) A diagram illustrating how C2B-3-4 is produced. Left panel: pictorial illustration of C2B-3-4 expression construct pC2B-3-4. A pair of complementary sequences are shown in short colored arrows. TSS, transcription start site; T, terminator; long blue arrow, promoter; boxes, exons; black circle, plasmid backbone; thicken green lines, flanking intronic sequence; black broken lines, backsplicing event; stacked lines, base pairing. Middle panel: duplex structure formed by transcribed RNA via base pairing. A 20 nt-long intronic sequence is given, along with the marked 5′ss and 3′ss, respectively. Sequences in red have non-*SMN* origin (see Methods and Materials, and Supplementary Figure S1 for more details). Right panel: diagrammatic representation of the overexpressed circRNA of interest. (**B**) Identification of circular transcripts generated from pC2B-3-4 expression vector. A representative gel showing circRNA of interest by using a pair of divergent primers targeting *SMN* exon 3. HeLa cells were transfected with or without pC2B-3-4 plasmids (in triplicate). Control refers to transfection with Lipofectamine-2000 alone. The size marker (M) is indicated on the left side of gel, while the identity of the band is indicated on the right side. The identity was confirmed by cloning and sequencing. (**C**) Quantification of C2B-3-4 expression levels by qPCR. Left panel: a diagrammatic representation of circRNA of interest. Annealing sites of primers are indicated. Middle panel: the expression of C2B-3-4 at the indicated concentrations. Right panel: the expression of C2B-3-4 at the indicated time point post transfection. Error bars represented standard error of the mean. (**D**) Identification of non-*SMN* circular transcripts generated from the pCGFP and pCHIPK2 expression vectors. Left panel: a diagrammatic representation of the expression vectors. Labeling is similar to (**A**). Right panel: a representative gel showing the expression of non-*SMN* circular transcripts. Labeling is the same as in (**B**). (**E**) Splicing of endogenous linear transcripts of *SMN* detected by a multi-exon skipping detection assay (MESDA) (see Methods and Materials for more details). Left panel: annealing positions of the primers used for MESDA are indicated. A representative gel showing the splicing pattern of the endogenous *SMN* genes in transfected cells, as determined by MESDA. Transfection type is indicated at the top of the gel. The size marker (M) is indicated on the left side of gel, while band identities are on the right side. “∆” specifies exon skipping. Right panel: bar diagram showing the relative expression of the individual *SMN* splice isoforms detected by MESDA as percent of total. An unknown band is indicated by “&”. Abbreviations: F, forward primer; R, reverse primer.

Abbreviations used in this publication are listed in Supplementary Table S1. Expression vectors constructed in this study are listed in Supplementary Table S2. Primers used in this study are listed in Supplementary Table S3. The pC2B-3-4 plasmid was constructed by inserting PCR amplified products in pCI vector (Promega, Madison, WI, USA, Cat No. E1731) using cDNA and/or genomic DNA as template, as described in Supplementary Figure S1. Other expression vectors were generated following the similar protocol using pC2B-3-4 as template (Figure 2, Supplementary Figure S2A and Supplementary Table S2). Briefly, pC2B-3-4 was digested with BsiWI and EcoNI to serve as backbone. Then, PCR products were digested with same enzymes and inserted between BsiWI and EcoNI sites. We used pcDNA5/FRT/TO (Thermo Fisher Scientific, Cat No. V652020) as backbone to make constructs in Flp-In™ system (Thermo Fisher Scientific), To generate expression vectors for stable cell lines, we amplified sequences between XhoI and NotI sites from pCI-based constructs and replaced XhoI with BamHI. Subsequently, inserts and pcDNA5/FRT/TO vector were digested by BamHI and NotI, and ligated through these two sites. The pcDNA5/FRT/TO-based vectors include pTC2A-2B-3-4^Int^, pTL2A-2B-3-4^Int^, pTC2B-3-4^Int^, pTL2B-3-4^Int^, pTC3-4^Int^ and pTL3-4^Int^ (Supplementary Table S2).

### 2.3. Establishment of Inducible Cell Lines

Cell lines established in this study are listed in Supplementary Table S4. T-REx™-293 cell line (Thermo Fisher Scientific, R71007, hereinafter referred as T-REx) was cultured in DMEM (Gibco, Cat No. 11965-092), supplied with 10% FBS (Gibco, Cat No. 16000-044) and 1× Glutamax (Gibco, Cat No. 35050061). All cell culture media and reagents were purchased from Life Technologies. Antibiotics including zeocin, blasiticidin, hygromycin (Life technologies Cat No. R250-01, R210-01, R220-05) and doxycycline (Dox) (Sigma-Aldrich, St. Louis, MO, USA, Cat No. D9891-10G) were used as instructed. Plasmids pOG44 and pcDNA5/FRT/TO were obtained from Thermo Fisher Scientific (Cat No. V600520 and Cat No. V652020). To establish stable cell lines for inducible expression of circRNAs of interest, the circRNA-expression plasmid was mixed with pOG44 at a 9:1 ratio (*w/w*). Then, these two plasmids were co-transfected into the host T-REx cell line by using Lipofectamine 2000 as per the manufacturer’s instructions. Of note, The Flp recombinase was expressed from pOG44, which facilitated integration of circRNA-expression plasmid into the host cell line. To estimate the transfection and integration efficacy, pcDNA5_FRT_mCH was used as a positive control (Addgene, Watertown, MA, USA, Cat No. 127109). Fresh medium was added 6 h (h) post transfection. At 24 h post transfection, cells were trypsinized and seeded at a density of 1.1 × 10^6^ cells per 100 mm dish. The concentration of hygromycin used to select for stable transfectants was determined to be 100 µg/mL. Medium containing hygromycin was replaced every 3 days. After 2 weeks of growth with hygromycin, the surviving cell colonies were isolated using cloning disks (Bel-ART, Wayne, NJ, USA, Cat No. 37847-0001) following the manufacturer’s instructions. To induce the expression of circRNA of interest, 0.1 µg/mL Dox was added to cell culture medium, and cells were collected for RNA preparation 24 h post treatment.

### 2.4. Transfection

Reverse transfection was used for transient gene overexpression. Unless stated otherwise, for each reverse transfection, cell suspension containing 0.25 × 10^6^ HeLa cells was mixed with pre-formed complexes between the plasmid DNA (0.4 µg) and Lipofectamine 2000 (1 µL) in a total volume of 0.5 mL. The mixture was then placed into a well of a 24-well plate. The medium was changed after 6 h. The cells were collected at 24 h post transcription.

### 2.5. Cytoplasmic and Nuclear Fractionation

The cytoplasmic and nuclear fraction were prepared using NE-PER™ Nuclear and Cytoplasmic Extraction Reagents (Thermo Fisher, Cat No. 78833) following the manufacturer’s instructions. CERI and NER buffers from the kit were supplemented with 1× Halt™ Protease and Phosphatase Inhibitor Cocktail (Thermo Scientific, 100X, Cat No. 78440) and 0.25–1 U/µL RiboLock RNase Inhibitor (Thermo Scientific, Cat No. EO0381). Around 5 × 10^6^–8 × 10^6^ cells were used for each fractionation. Cells were collected by trypsinization, pelleted and washed twice with ice-cold Dulbecco’s phosphate-buffered saline (DPBS, Gibco, Cat No. 14190-144).

### 2.6. Western Blot

Protein concentration was measured by Bio-Rad protein assay (Bio-Rad, Hercules, CA, USA, Cat No. 5000006). A total amount of 8 µg of protein was loaded per each lane of a 10% SDS-PAGE gel. After electrophoresis, proteins were transferred to PVDF membranes using a Transblot Turbo fast transfer system (Bio-Rad). Membranes were blocked in 5% non-fat milk dissolved in Tris-buffered saline containing 0.05% Tween-20 (TBST). Primary antibody incubation was carried out at 4 °C overnight. Primary antibody dilutions were as follows: mouse anti-α-tubulin 1:4000 (Sigma-Aldrich, Cat No. T6199), rabbit anti-Histone H3 1:4000 (Abcam, Waltham, MA, USA, Cat No. 1791). After incubation with primary antibodies, membranes were washed in TBST three times and incubated with secondary antibodies for 1 h at room temperature. Secondary antibodies and their dilutions were as follows: goat anti-mouse 1:4000 (Jackson ImmunoResearch Laboratories Inc, West Grove, PA, USA, Cat No. 115-035-003), donkey anti-rabbit 1:2000 (Cytiva USA, Marlborough, MA, USA, Cat No. NA934). After secondary antibody incubations, membranes were washed in TBST again and developed using SuperSignal West Femto Maximum Sensitivity Substrate (Thermo Scientific, Cat No. 34094). Bands were then visualized using a UVP Biospectrum AC imaging system.

### 2.7. RNA Isolation and RNase R Treatment

Total RNA was isolated from cells using TRIzol reagent (Invitrogen, Waltham, MA, USA, Cat No. 15596018). RNA was treated with RQ1 RNase-free DNase (Promega, Cat No. M6101), followed by phenol:chloroform extraction and ethanol precipitation. RNase R treatment was carried out in 10 µL reaction, in which 2 µg of RNA was digested by 0.5 µL (10 U) of RNase R (Applied Biological Material Inc., Richmond, BC, Canada, Cat No. E049). Of note, RNA samples used in the RNase R treatment were treated with DNase first. The treatment was performed for 45 min (min) at 37 °C, followed by the enzyme inactivation at 65 °C for 20 min. To assess the efficiency of digestion, mock reactions in which the enzyme was omitted were performed side by side.

### 2.8. Reverse Transcription and PCR (RT-PCR)

cDNA was synthesized using Superscript III reverse transcriptase (RT) (Invitrogen), following the manufacturer’s instructions. Specifically, 0.5 µg of total RNA was used per 5 µL RT reaction, employing either a gene-specific (Supplementary Table S3) or a random primer (Promega, Cat No. C1181). For semi-quantitative PCR, 1 µL of cDNA reaction was used as a template in 20 µL reaction. PCR products were separated on 5.3% or 6% native polyacrylamide gels and visualized by ethidium bromide staining. For qPCR, cDNA was first diluted 20 times, and 3 µL was used as a template per 20 µL qPCR reaction containing 1× PowerUp SYBR green master mix (Life Technologies, Cat No. A25742) and the desired pair of primers. qPCR reactions in biological or technical triplicates were performed on a QuantStudio 3 (Thermo Fisher) thermocycler. Relative expression was determined using the 2^-ΔΔCt^ method using GAPDH as the normalizing gene for gene expression assays. To determine the copy number of circRNA per cell, we employed standard curves that were generated using absolute amounts of the linearized plasmids containing the sequence of the expected PCR products amplified from circRNA of interest. As per published reports, a single HeLa and HEK 293 cell contain ~30 pg and ~16 pg RNA, respectively (https://www.thermofisher.com/us/en/home/references/ambion-tech-support/rna-tools-and-calculators/macromolecular-components-of-e.html (accessed on 4 January 2022), https://www.aatbio.com/resources/faq-frequently-asked-questions/What-is-the-total-RNA-content-in-HeLa-cells#:~:text=For%20HeLa%20cells%2C%20the%20total,30%20pg%20per%20HeLa%20cell (accessed on 4 January 2022), https://www.miltenyibiotec.com/_Resources/Persistent/ca9f513c68ed01981bc4d7aa25b01c90db75e6f5/Average_RNA_yields.pdf (accessed on 4 January 2022)). We confirmed RNA concentration per cell in the range of the reported concentrations. We used 0.015 µg RNA to synthesize cDNA for each qPCR. To obtain the copy number per cell, the quantified total copy number in each qPCR reaction was divided by the cell number. Error bars represent standard error of the mean. Statistical significance: *, *p* < 0.05; **, *p* < 0.01. All primers were obtained from Integrated DNA Technologies.

### 2.9. Multi-Exon Skipping Detection Assay (MESDA)

MESDA is a powerful technique developed in 2012 to determine the relative abundance of the alternative spliced transcripts of *SMN* [27]. As many as 16 alternatively spliced variants of *SMN* have been detected in a single reaction using MESDA [28]. While technique does not distinguish between transcripts of *SMN1* and *SMN2*, it bridges a critical gap that cannot be reliably filled by available methods, including qPCR. Only exon 7 is differently regulated between *SMN1* and *SMN2* genes, and there is a DdeI digestion method to capture those differences [27]. However, when it comes to determining the relative abundance of the vast number of alternatively spliced transcripts, as far as *SMN* are concerned, MESDA remains the method of choice. Of note, conclusions of MESDA rely upon the comparison of different bands within the same lane. Hence, findings of MESDA cannot be altered by differential loading of samples or changing the nature of control in the neighboring lane. We have confirmed the identity of each band detected by MESDA by sequencing. Method has an inherent advantage to provide useful information even when a single sample is analyzed without any control, particularly when the primary goal is to determine the relative abundance of the alternatively spliced transcripts of *SMN*. We performed MESDA using forward and reverse primers at exons 1 and 8, respectively [28,29]. The cDNA was prepared as described in the RT-PCR section. To ensure capturing of the low abundant spliced variants of *SMN*, we performed MESDA using the 5′-end-P32-radio-labelled primer, 3′ Ex8-25, as reported earlier [29].

### 2.10. Identification of Circular and Linear Transcripts

Bands of interest corresponding to RT-PCR products were excised from native polyacrylamide gels, and DNA was eluted using a “crush and soak” method. The recovered DNA was then cloned into a pGEM-T Easy vector (Promega, Cat No. A1360) following the manufacturer’s instructions. After initial screening by blue/white colony selection on indicator plates, colonies were subjected to a colony PCR. Colonies with plasmids that carried inserts of the expected size were propagated, and plasmids purified using the QIAprep Spin Miniprep Kit (Qiagen, Germantown, MD, USA, Cat No. 27014). Insert identities were determined by Sanger sequencing.

### 2.11. Statistical Analysis

All calculations were performed in Excel (Microsoft, Version 16.62, Redmond, WA, USA). Data were expressed as mean ± standard error of the mean (SEM). Statistical analyses were performed using the unpaired Student’s *t*-test. Unless otherwise mentioned, experiments were performed in triplicate, *p* values were two-tailed and the level of statistical significance was set as *p* < 0.05.

## 3. Results

### 3.1. Expression of a SMN circRNA from an Engineered Vector

We generated a pC2B-3-4 expression vector to overexpress C2B-3-4, one of the most abundant *SMN* circRNAs encompassing exons 2B, 3 and 4. Of note, pC2B-3-4 lacked intervening intronic sequences (Figure 2A). For circRNA production, we included flanking sequences of introns 2A and 4 with their corresponding 3′ss and 5′ss upstream and downstream of exons 2B and 4, respectively. We also added the inverted repeat sequences to create an RNA:RNA duplex expected to facilitate splice site pairing needed for backsplicing (Figure 2A and Supplementary Figure S1). We examined the expression of C2B-3-4 in HeLa cells 24 h post transfection. To do so, we first treated RNA samples with RNase R, which selectively degrades linear transcripts. We then analyzed circRNA overexpression by RT-PCR using divergent primers that annealed to *SMN* exon 3, as previously described [25]. We observed robust amplification of C2B-3-4 in cells transfected with pC2B-3-4 when we used 22 cycles of PCR (Figure 2B). As expected, at this low number of cycles, we did not detect endogenous C2B-3-4 in the untransfected cells (Figure 2B). We confirmed the identity of the PCR product by cloning and sequencing. To examine the relationship between pC2B-3-4 concentration and the level of C2B-3-4 overexpression, we transfected HeLa cells with the increasing amounts of the plasmid for 24 h. We detected expression of C2B-3-4 at all plasmid concentrations used, with the highest expression observed at the highest plasmid concentration employed (Figure 2C). We then used the highest plasmid concentration to determine the time at which C2B-3-4 was most abundantly expressed following transfection (Figure 2C). We collected cells at different time points, starting with 12 h post transfection. As shown in Figure 2C, the highest expression of C2B-3-4 was observed at 24 h post transfection, followed by a steep decline.

To demonstrate that our approach to produce C2B-3-4 circRNA is universally applicable, we constructed pCGFP and pCHIPK2 plasmids to overexpress circRNAs from non-mammalian *eGFP* and human *HIKP2* exon 2, respectively (Figure 2D). Of note, CGFP is an artificial circRNA, while circRNA generated from exon 2 of *HIPK2* is reported to be an abundant circRNA involved in astrocyte activation [30]. We observed expression of both non-*SMN* circRNAs at the levels comparable to that of C2B-3-4, validating that our approach to generate circRNAs could be applied universally (Figure 2D). Our results also confirm that the strength of the splice sites and the RNA:RNA duplex formed between the inverted repeats could serve as the major drivers of backsplicing regardless of exonic sequences. The human *SMN* genes produce multiple alternatively spliced isoforms of *SMN* [31]. MESDA has been used as a powerful technique to determine the relative abundance of linear *SMN* splice isoforms in a single experiment [27,29,32]. Exon 3 of *SMN* contains a complementary sequence with the potential to form an RNA:RNA duplex between two *SMN* transcripts and affect the relative abundance of the alternatively spliced transcripts of *SMN* [25]. We employed MEDSA to determine whether C2B-3-4 overexpression perturbs the relative abundance of linear transcripts generated by the endogenous *SMN*. As controls, we used the non-*SMN* circRNAs overexpressed from pCGFP and pCHIPK2. We observed no appreciable effect on splicing of the *SMN* genes for any of the three circRNAs (Figure 2E). These results suggest that the overexpression of C2B-3-4 or pCGFP or pCHIPK2 does not perturb the structural context of pre-mRNAs generated from the endogenous *SMN* to affect the outcome of splicing of endogenous *SMN* transcripts.

### 3.2. Effect of Internal Introns on Expression of SMN circRNAs

**Figure 3:**
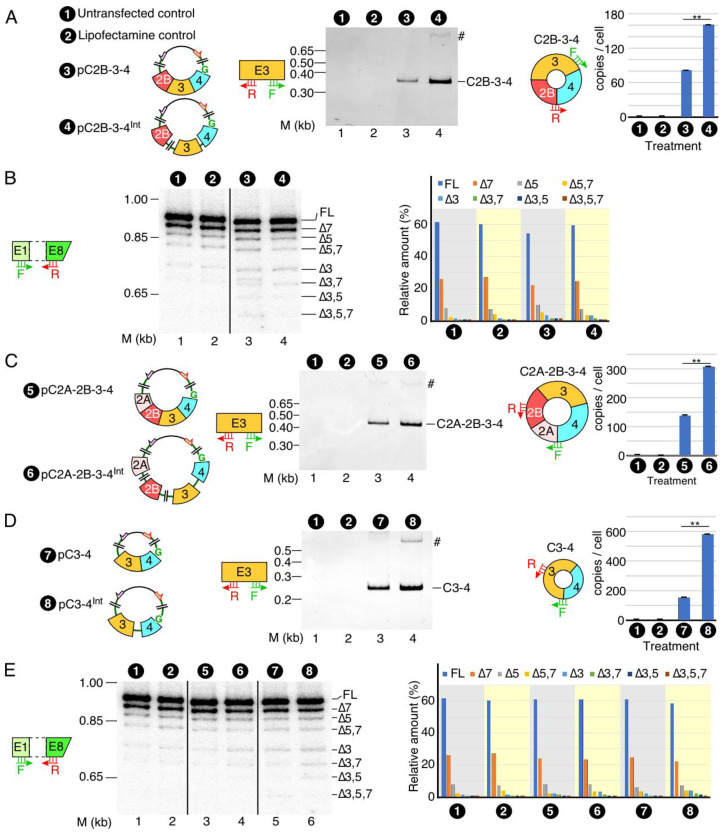
The effect of internal introns on the expression of *SMN* circRNAs. (**A**) The expression of C2B-3-4. Left panel: diagrammatic representation of plasmid used to overexpress C2B-3-4. Middle panel: a representative gel showing the results of semi-quantitative PCR with divergent primers annealing to exon 3. The type of treatment is specified at the top of the gel. The size marker (M) is indicated on the left side of gel, while the identity of the band is indicated on the right side. A band likely derived from duplicate of indicated circular transcript is marked by “#”. Right panel: bar diagram showing the copy number of C2B-3-4 per cell, as determined by qPCR. Annealing positions of primers used are indicated. Error bars represent standard error of the mean. Statistical significance: **, *p* < 0.01. Abbreviations are the same as in Figure 2. (**B**) Splicing of endogenous linear transcripts of *SMN* detected by MESDA. Left panel: a representative gel showing the splicing pattern of the endogenous *SMN* genes in transfected cells, as determined by MESDA. Labeling is the same as in Figure 2E. Right panel: bar diagram showing the relative expression of the individual *SMN* splice isoforms detected by MESDA as precent of total. Labeling is the same as in Figure 2E. (**C**) The expression of C2A-2B-3-4. Left panel: diagrammatic representation of overexpressed circRNAs, along with their number coding. Middle panel: a representative gel showing the results of semi-quantitative PCR. Labeling is the same as Figure 3A. Right panel: bar diagram showing the copy number of C2A-2B-3-4 per cell, as determined by qPCR. Annealing positions of primers used are indicated. Error bars represent standard error of the mean. Statistical significance: **, *p* < 0.01. (**D**) The expression of C3-4. Left panel: diagrammatic representation of overexpressed circRNAs, along with their number coding. Middle panel: results of semi-quantitative PCR. Labeling is the same as in Figure 3A. Right panel: bar diagram showing the copy number of C3-4 per cell, as determined by qPCR. Annealing positions of primers used are indicated. Error bars represent standard error of the mean. Statistical significance: **, *p* < 0.01. (**E**) Left panel: a representative gel showing the splicing pattern of the endogenous *SMN* genes in transfected cells, as determined by MESDA. Labeling is the same as in Figure 2E. Right panel: bar diagram showing the relative expression of the individual *SMN* splice isoforms detected by MESDA as percent of total. Labeling is the same as in Figure 2E.

To determine the effect of internal introns on the expression of C2B-3-4, we constructed pC2B-3-4^Int^, in which wild-type introns 2B and 3 were inserted after exons 2B and 3, respectively (Figure 3A). To reduce the size, intron 2B had an internal deletion. Of note, both pC2B-3-4^Int^ and pC2B-3-4 code for the identical circRNA, C2B-3-4. However, pre-mRNA generated from pC2B-3-4^Int^ harbors introns 2B and 3, whereas pre-mRNA generated from pC2B-3-4 lacks introns 2B and 3. In order to generate C2B-3-4 from pC2B-3-4^Int^, introns 2B and 3 present within pre-mRNA must be spliced out by forward splicing. On the other hand, generation of C2B-3-4 from pC2B-3-4 does not require any intron removal by forward splicing. We transfected HeLa cells with pC2B-3-4^Int^ and pC2B-3-4 and compared the expression levels of C2B-3-4 generated from these plasmids. We observed a more than two-fold increase in the expression of C2B-3-4 from pC2B-3-4^Int^ as compared to pC2B-3-4, supporting the positive impact of internal introns on the expression of this circRNA (Figure 3A). We did not observe larger bands corresponding to circRNAs with retained introns 2B and/or 3 of *SMN* (Figure 3A). These results suggest that the intercalating introns are efficiently removed either before or after backsplicing. We employed MESDA to determine if the transcripts produced from pC2B-3-4^Int^ influenced splicing of pre-mRNA generated from the endogenous *SMN*. We observed no effect of overexpression of C2B-3-4 or its intron-containing precursors on the splicing of transcripts generated from the endogenous *SMN* (Figure 3B). Once again, these results underscore that the overexpression of C2B-3-4 or its intron-containing precursors do not perturb the structural context of pre-mRNAs generated from the endogenous *SMN* to affect the outcome of splicing of endogenous *SMN* transcripts.

Similar to C2B-3-4, C2A-2B-3-4 and C3-4 are abundantly expressed circRNAs of *SMN*. To overexpress C2A-2B-3-4, we generated intronless pC2A-2B-3-4 and intron-containing pC2A-2B-3-4^Int^ (Figure 3C). For overexpression of C3-4, we generated intronless pC3-4 and intron-containing pC3-4^Int^ constructs (Figure 3D). We then compared the expression levels of C2A-2B-3-4 and C3-4 in HeLa cells transfected with both intronless and intron-containing vectors. Just like in the case of C2B-3-4, we observed a more than two-fold increase in the expression of C2A-2B-3-4 and C3-4 when their expression vectors harbored internal introns. These results indicate that the stimulatory effect of introns on backsplicing did not depend on the number introns or exons in the precursors of the circRNA-generating transcripts. We performed MESDA to determine if the overexpression of C2A-2B-3-4 or C3-4 influenced the relative expression of linear splice isoforms generated from the endogenous *SMN*. We observed no effect of overexpression of C2A-2B-3-4 and C3-4 on the splicing of transcripts generated from endogenous *SMN* (Figure 3E). These results underscore that the overexpression of C2A-2B-3-4 and C3-4 or their intron-containing precursors do not perturb the structural context of pre-mRNAs generated from the endogenous *SMN* to affect the outcome of splicing of endogenous *SMN* transcripts.

In order to compare and contrast the expression of the linear counterparts to our circRNA constructs, we constructed expression vectors to generate linear transcripts L2A-2B-3-4, L2B-3-4 and L3-4 corresponding to circRNAs C2A-2B-3-4, C2B-3-4 and C3-4, respectively, by inserting a single G-to-C mutation (G1C substitution) at the 5′ss of intron 4. We made these constructs in the context of both intronless and internal-intron-containing sequences. As expected, G1C substitution fully inhibited production of circRNAs of interest in all cases (Supplementary Figure S2C). We also examined the levels of linear transcripts produced from the *SMN* circRNA-generating vectors. We observed that all three of these vectors made linear transcripts as well, although their levels were lower than those generated by the corresponding vectors carrying G1C substitutions (Supplementary Figure S2D). The alternatively spliced exon 3 is an internal exon in the intron-containing precursor transcripts for C2A-2B-3-4 circRNA, as well as L2A-2B-3-4, L2B-3-4 and C2B-3-4 RNAs. We detected skipping of exon 3 in all the linear transcripts. Interestingly, linear transcripts generated from the circRNA expression vectors pC2A-2B-3-4 and pC2B-3-4 had a higher degree of exon 3 skipping than transcripts L2A-2B-3-4 and L2B-3-4, respectively (Supplementary Figure S2D). These results support that abrogation of the 5′ss of exon 4 has as a stimulatory effect on inclusion of exon 3. Of note, some of the differences in the levels of *SMN* transcripts (linear or circular) could be due to differential stability of linear and/or circular transcripts or their precursors. However, uncovering mechanisms that determine the turnover rate of specific transcripts of *SMN* is beyond the scope of this study.

### 3.3. Efficiency of Generation of a SMN circRNA from a Single Chromosomal Location

**Figure 4:**
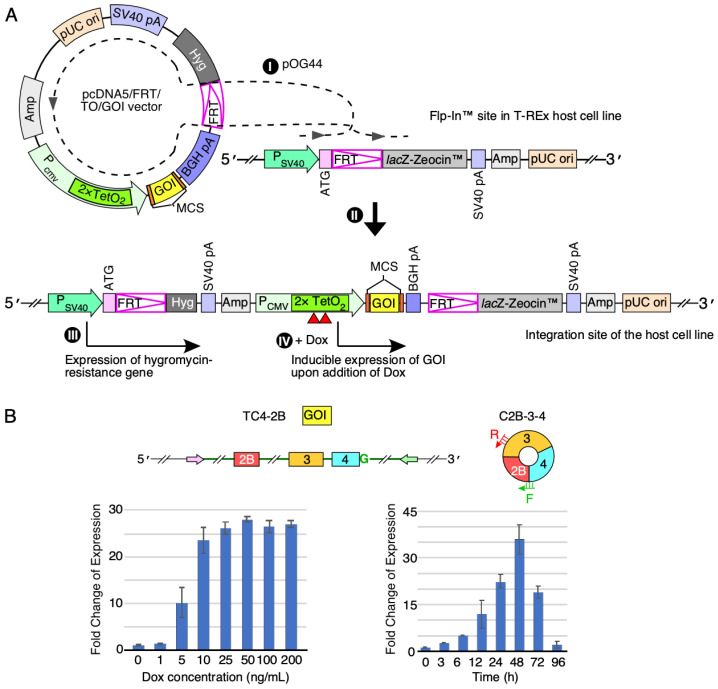
Establishment of inducible stable cell lines to overexpress *SMN* circRNAs. (**A**) Left panel: diagrammatic representation of Flp-In system used to generate a stable cell line with inducible expression of a gene of interest (GOI) (edited from https://www.thermofisher.com/order/catalog/product/V652020#/V652020 (accessed on 15 January 2021)). (I) Co-transfection of pcDNA5/FRT/TO/GOI vector (expression vector for a GOI) and pOG44 (expression vector for Flp recombinase) into a host cell line that contains an FRT (Flp recombination target) site at a specific location in the genome; (II) integration of pcDNA5/FRT/TO/GOI vector at the FRT site mediated by Flp recombinase; (III) selection for cells resistant to hygromycin (resistance is provided by stably integrated pcDNA5/FRT/TO/GOI); (IV) induction of GOI expression by doxycycline (Dox) and analysis. Dox addition is indicated by red triangles. The diagram is not to scale. Relevant abbreviations: GOI, gene of interest; MCS, multiple cloning site; *Hyg*, hygromycin-resistance gene; *Amp*, ampicillin-resistance gene; TetO_2_, tetracycline operator, Dox, doxycycline. (**B**) Establishing the optimal regiment, including Dox dosage and induction duration, for C2B-3-4 expression in the stable cell line we called TC4-2B. Left panel: bar diagram showing the fold change of C2B-3-4 expression in response to different Dox concentrations (ng/mL). For fold change, the expression level at 0 ng/mL was normalized to a value 1. Right panel: bar diagram showing the fold change of C2B-3-4 expression in response to different duration of Dox treatment. For fold change, the expression level at 0 h was normalized to a value 1. In addition, diagrammatic representations of the gene of interest used for overexpression, as well as C2B-3-4 itself, are given above the panels. Exonic sequences are represented by colored boxes; intronic by lines/broken lines. The pink and green arrows represent complementary regions used to promote backsplicing. “G” in green signifies the functional 5′ss of exon 4 required for backsplicing. Annealing positions of primers used for circRNA detection are indicated.

We next examined the feasibility and efficiency of *SMN* circRNAs expression when a circRNA-producing construct is stably integrated into the cellular genome at a single site. We performed this study by establishing a stable cell line in which overexpression of C2B-3-4 was switched on by doxycycline (Dox) using the commercially available FRT/TO system and modified HEK 293 cells (T-REx™-293 cells, hereinafter referred to as T-REx) that carry a Flp recombination target (FRT) site in their genome. The FRT/TO system allows incorporation of the exogenous gene of interest (expressed from pcDNA5/FRT/TO vector) at the FRT site assisted by Flp recombinase (expressed from pOG44 plasmid) (Figure 4A). We started with cloning of the internal-intron-containing C2B-3-4 sequence into the pcDNA5™/FRT/TO vector. We then transfected this vector together with pOG44 plasmid into T-REx cells. Using selectable markers, we randomly chose twenty colonies of stable transfectants (Supplementary Figure S3A). We analyzed five of these colonies for the expression of C2B-3-4 after the 24-h-treatment with three concentrations of Dox (100, 300 and 1000 ng/mL). According to the qPCR results, we observed a more than 10,000-fold increase of C2B-3-4 levels at all Dox concentrations (Supplementary Figure S3B). As expected, there was no effect of Dox on C2B-3-4 expression in the untransfected control (Ctrl). We used low Dox concentration (100 ng/mL) to screen the rest of the 20 colonies.

We selected a lead clone (colony 6, hereafter referred to as TC4-2B) to perform further characterization. TC4-2B displayed low baseline (leaky) expression of C2B-3-4 and higher fold change in expression of C2B-3-4 after induction with Dox. We then used a broad concentration range of the antibiotic to monitor expression of C2B-3-4 in TC4-2B cells (Figure 4B). We observed a dose-dependent increase in expression of C2B-3-4 in the Dox concentration range from 1 to 10 ng/mL. We chose a 5 ng/mL Dox treatment to determine the time at which C2B-3-4 was maximally expressed. We collected samples at eight time points, starting from 0 h to 96 h after the initiation of Dox treatment. We observed a progressive increase in C2B-3-4 levels with time until 48 h after single Dox induction (Figure 4B). At the peak of expression, we recorded a more than 30-fold increase in the C2B-3-4 levels compared to the background level of expression at the 0 h time point. Of note, the C2B-3-4 levels produced by TC4-2B cells were comparable to those observed with the transient transfection at the highest plasmid concentration we tested. Hence, we consider the expression level of C2B-3-4 from TC4-2B cells as robust given the fact that transcripts were generated from a single transgene inserted within the genome compared to potentially thousands of copies of plasmid within a single nucleus in the case of transient transfection [33]. The half-life of Dox was reported as ~48 h at 37 °C [34,35]. Consistently, we observed a substantial drop in the C2B-3-4 expression level after 48 h of Dox treatment.

### 3.4. Comparative Analysis of SMN circRNAs Generated in Stable Cell Lines

**Figure 5:**
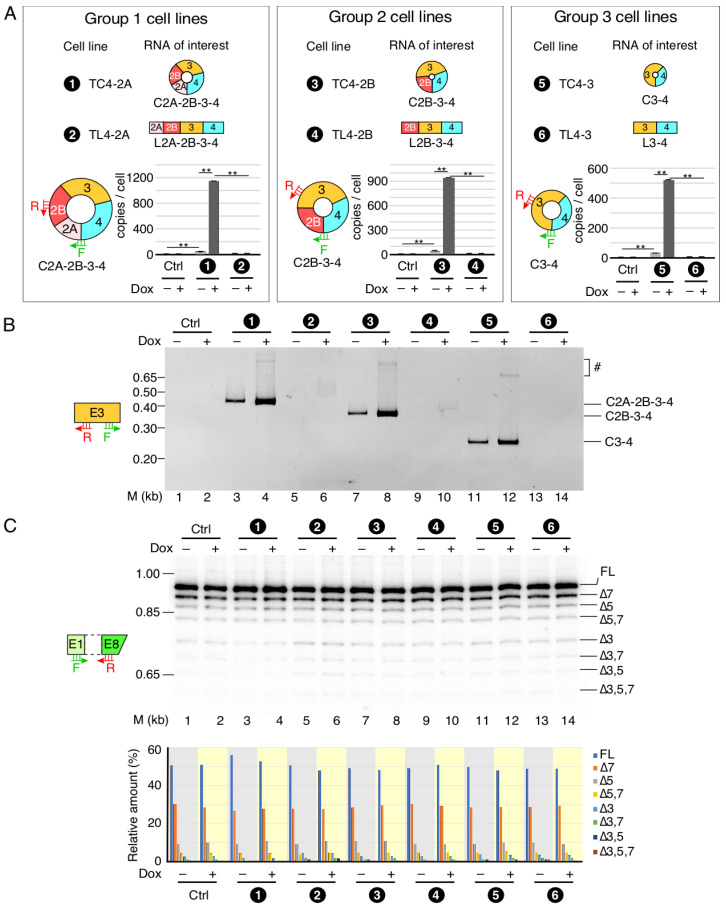
Comparative analysis of *SMN* RNA expression in stable cell lines. (**A**) Inducible expression of circRNA of interest in the established cell lines. Based on the number of exons in circular or linear transcripts, three groups of cell lines are shown. The names of each stable cell line are given as well, along with their number coding. Number coding used is as follows. (1) TC4-2A, stable cell line expressing C2A-2B-3-4 circRNA. (2) TL4-2A, stable cell line expressing LC2A-2B-3-4 linear RNA produced due to G1C mutation at the 5′ss of exon 4. (3) TC4-2B, stable cell line expressing C2B-3-4 circRNA. (4) TL4-2B, stable cell line expressing L2B-3-4 linear RNA produced due to G1C mutation at the 5′ss of exon 4. (5) TC4-3, stable cell line expressing C3-4 circRNA. (6) TL4-3, stable cell line expressing L3-4 linear RNA produced due to G1C mutation at the 5′ss of exon 4. Bar diagrams show the copy number of the indicated circRNAs in the presence (+) or absence (−) of Dox, as determined by qPCR. Annealing positions of primers used are indicated. The original T-REx cell line was used as a control (Ctrl). Error bars represent standard error of the mean. Statistical significance: **, *p* < 0.01. (**B**) A representative gel showing the results of semi-quantitative PCR for circRNA detection using divergent primers annealing to exon 3. Numbers at the top of the gel indicate stable cell lines as described in panel (**A**). Ctrl, control cell line. (+) and (−) represent presence and absence of Dox, respectively. The size marker (M) is indicated on the left side of gel, while the identity of the bands is indicated on the right side. The bands likely derived from duplicates of indicated circular transcript are marked by “#”. (**C**) Relative expression of endogenous *SMN* transcripts detected by MESDA. Top panel: a representative gel showing the splicing pattern of the endogenous *SMN* genes in stable cell lines, as determined by MESDA. Cell lines are number-coded as described in panel (**A**). Ctrl, control cell line. (+) and (−) represent presence and absence of Dox, respectively. The size marker (M) is indicated on the left side of gel, while the identity of the bands is indicated on the right side. Bottom panel: bar diagram showing the relative expression of the individual *SMN* splice isoforms detected by MESDA as precent of total. Cell lines and the presence (+) or absence (−) of Dox are indicated at the bottom. The rest of labeling is the same as in Figure 2E.

Following the protocol employed for establishing the TC4-2B cell line, we generated five additional cell lines to overexpress other *SMN* circular and linear transcripts. Two of these cell lines, TC4-2A and TC4-3, were designed to overexpress *SMN* circRNAs C2A-2B-3-4 and C3-4, respectively (Figure 5A). The other three cell lines were TL4-2A, TL4-2B and TL4-3, which were designed to overexpress linear *SMN* transcripts L2A-2B-3-4, L2B-3-4 and L3-4, respectively (Figure 5A). For each cell line, we screened between 11 and 20 clones and selected a lead candidate for further study using the same criteria we used for TC4-2B (Figure 5A). We analyzed both circular and linear transcripts by PCR using divergent and convergent primer combinations, respectively (Figure 5B and Supplementary Figure S4). We observed some level of expression of linear transcripts that “escaped” circularization in all the stable cell lines designed to overexpress the circRNAs (Supplementary Figure S4). Importantly, these levels were lower compared to the levels detected in the cell lines designed to overexpress the linear *SMN* transcripts. L3-4 was an exception as its level in TC4-3 appeared to be the same as in TL4-3 (Supplementary Figure S4).

The TL4-2A cell line programmed to synthesize linear transcript L2A-2B-3-4 could potentially generate smaller circRNAs using 5′ss of exon 2A or 2B or 3. However, divergent primers annealing to these exons failed to amplify smaller circRNAs from the TL4-2A cell line (Figure 5B, Supplementary Figure S5). Similarly, we did not detect smaller circRNAs in transcripts isolated from TL4-2B and TL4-3. Divergent primers annealing to exons 2A and 2B have the potential to amplify circRNAs with skipped exon 3. However, we were unable to detect any product corresponding to a circRNA with skipped exon 3, suggesting mechanistic constraint in the generation of these circRNAs. Similar to the results obtained with transient transfection, we observed linear transcripts lacking exon 3 in stable cell lines programmed to generate C2A-2B-3-4, L2A-2B-3-4, L2B-3-4 and C2B-3-4 (Supplementary Figure S4). We also used MESDA to determine whether overexpression of the *SMN* transgenes in our inducible cell lines would influence splicing of the endogenous *SMN* (Figure 5C). We observed no effect of induction on the splicing of endogenous *SMN* in inducible cell lines harboring *SMN* transgene. These results are consistent with those obtained with the transiently transfected cells overexpressing *SMN* circRNAs and support that stably expressing *SMN* circRNAs do not perturb the structural context of pre-mRNAs generated from the endogenous *SMN* to affect the outcome of splicing of endogenous *SMN* transcripts.

### 3.5. Localization of SMN circRNAs

**Figure 6:**
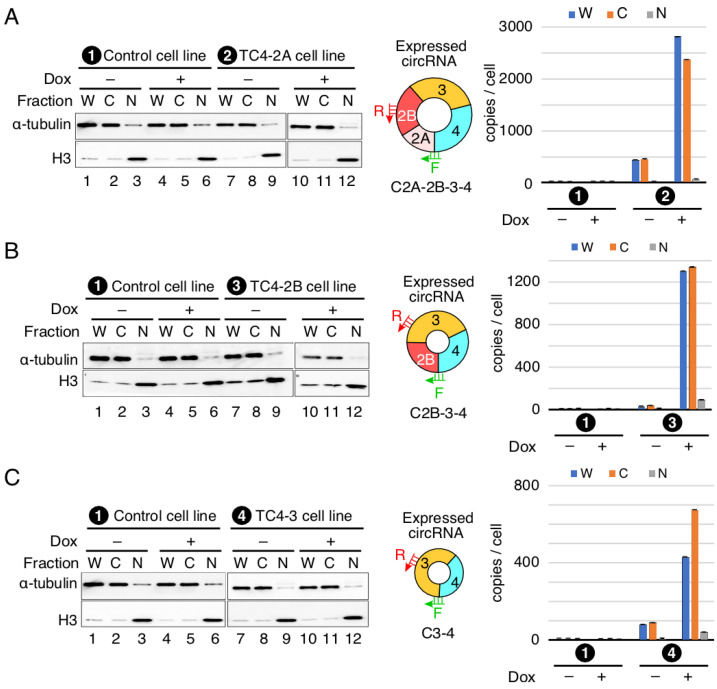
Cellular localization of *SMN* circRNAs. (**A**) Localization of C2A-2B-3-4. Left panel: diagrammatic representation of C2A-2B-3-4 expressed in TC4-2A stable cell line designated as (2). Middle panel: Western blot result showing the expression of α-tubulin and H3 in different cell fractions. Cell lines used are indicated at the top of the gels, with (+) and (−) signifying the presence and absence of Dox, respectively. Abbreviations: W, whole cell lysate; C, cytoplasmic fraction; N, nuclear fraction. Middle panel shows the type of circRNA overexpressed. Annealing positions of primers used for qPCR are shown. Right panel: bar diagram showing the copy number of C2A-2B-3-4 per cell, as determined by qPCR. Labeling of cell lines are same as in the left panel. (**B**) Localization of C2B-3-4. All the labeling is the same as in (**A**). (**C**) Localization of C3-4. All the labeling is the same as in (**A**).

We examined the nuclear and cytoplasmic distribution of *SMN* circRNAs in stable cell lines designed to overexpress *SMN* circRNAs upon induction with Dox. We used histone H3 (H3) and α-tubulin as markers to distinguish nuclear and cytoplasmic fractions, respectively. The results of the Western blot show the expected distribution of α-tubulin and H3 (Figure 6), confirming the optimal fractionation of cellular extracts we employed in our localization studies. The results of the qPCR using RNA prepared from the nuclear and cytoplasmic fractions show that overexpressed *SMN* circRNAs were localized in the cytoplasm (Figure 6). These findings are consistent with previous reports showing predominant cytosolic localization of the exon-only-containing circRNAs [36,37].

## 4. Discussion

The human *SMN* genes code for a vast repertoire of circRNAs. The most abundant of these circRNAs are generated by the first four internal exons, e.g., exons 2A, 2B, 3 and 4. However, the mechanism of their production remains unknown. We began this study with the construction of a mammalian expression vector that allowed overexpression of C2B-3-4, an *SMN* circRNA comprised of exons 2B, 3 and 4. The expression of C2B-3-4 was enabled by a strong RNA:RNA duplex that brought the downstream 5′ss of exon 4 in proximity with the upstream 3′ss of exon 2B, forcing backsplicing between these two splice sites (Figure 2A). Despite the absence of introns 2B and 3, the expression of C2B-3-4 from the vector was 1000-fold higher than the background level expression in the untransfected cells. Employing a similar approach, we generated two non-*SMN* circRNAs containing *eGFP* and *HIPK2* sequences. High expression of these non-*SMN* circRNAs suggested that backsplicing was primarily driven by the common RNA:RNA duplex that brought the splice sites together. The *SMN* genes generate multiple alternatively spliced transcripts due to skipping exons 3, 5 and 7 in different combinations [31]. We observed no effect of overexpression of any of the circRNAs on the relative abundance of splice isoforms generated from the endogenous *SMN*.

**Figure 7:**
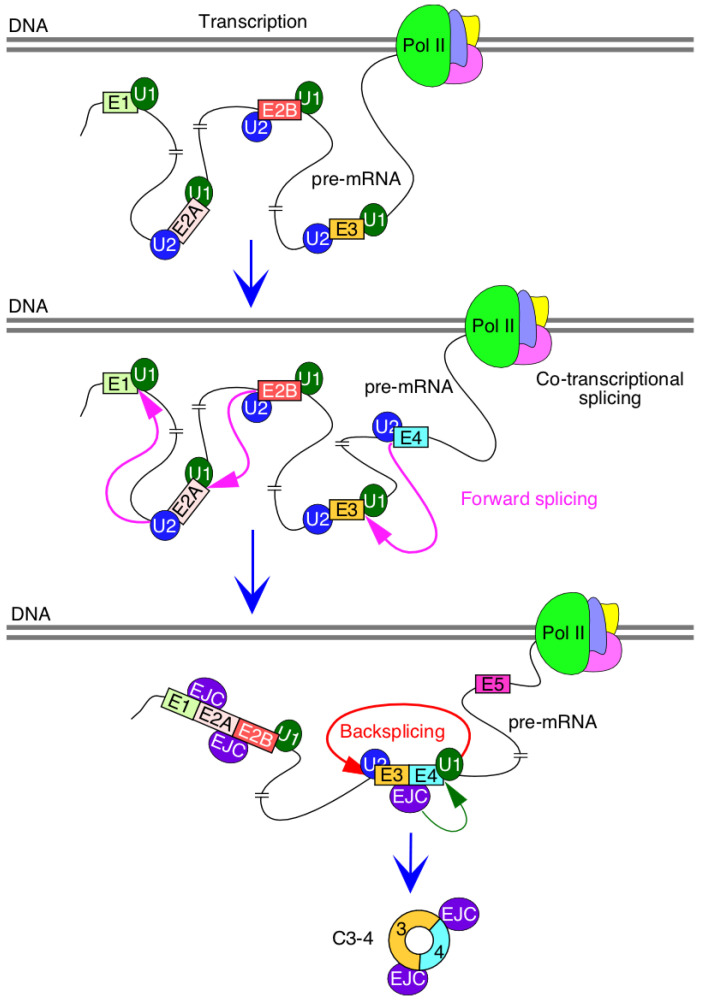
Mechanism of *SMN* circRNA generation. Splicing factors and U snRNP are recruited during transcription by RNA polymerase II (pol II). Many introns could be potentially removed by forward splicing while pol II is still transcribing. Removal of introns is accompanied by deposition of exon junction complex (EJC) at the exon-exon junction. In some instances, EJC may help recruit splicing factors and promote splicing of neighboring introns. We hypothesize that EJC deposited at the junction of exons 3 and 4 promotes backsplicing involving the 5′ss of exon 4.

Production of circRNAs by backsplicing requires an efficient pairing of a downstream 5′ss with an upstream 3′ss. In the case where multiple exons are located within a circRNA, removal of intron(s) by forward splicing is required so that an RNA circle is fully devoid of an intronic sequence. It is generally difficult to establish if the removal of an intron by forward splicing precedes or follows the backsplicing event. However, it is possible to test the proof of principle that forward splicing of introns within the circularized region modulates the backsplicing event. We tested this proof of principle in the context of the three abundantly expressed exon-only circRNAs of *SMN*, C3-4, C2B-3-4 and C2A-2B-3-4, which require removal of 1, 2 and 3 introns, respectively. All three circRNAs use 5′ss of exon 4 but different 3′ss. We observed noticeable upregulation of circRNAs upon insertion of intervening introns, suggesting that factors recruited to the internal introns expedite the backsplicing event. Multiple mechanisms may account for this stimulatory effect, including a better recruitment of U1 snRNP at the 5′ss of exon 4, promoted by cross-exon interactions initiated by factors recruited at the 3′ss of exon 4. Such a mechanism will be consistent with the exon definition model in which recognition of splice sites at each end of an internal exon is promoted by a network of factors enveloping the entire exon [38]. Transcription is coupled to splicing, and removal of intronic sequences is accompanied by the deposition of the exon junction complex, EJC, which is known to promote splicing of the neighboring introns [39,40] (Figure 7). Moreover, eukaryotic initiation factor eIF4A3, one of the components of EJC, has been implicated in circRNA generation [41]. Hence, it is possible that components of EJC, including eIF4A3, are regulators of *SMN* circRNA generation (Figure 7). A mutually inclusive hypothesis would be that the intercalating introns increase the size of precursor transcripts, rendering more flexible structures and facilitating backsplicing. Future studies will reveal if these and additional mechanisms that disproportionately confer enhanced stability of circRNAs contribute towards the elevated levels of circRNAs produced by the intron-containing precursors.

We established the inducible stable cell lines for overexpression of C3-4, C2B-3-4 and C2A-2B-3-4 upon induction with Dox. Unlike transient transfections in which multiple copies of the expressing vector could be present within a single cell, stable cell lines incorporated a single transgene of interest at a specific site within the genome. All these stable cell lines designed to express *SMN* circRNAs harbored internal introns. We observed robust expression of *SMN* circRNAs upon induction with Dox. As in the case of transient transfections, we observed no effect of the overexpressed *SMN* circRNAs on splicing of the endogenous *SMN* in any of the stable cell lines. These results support that factors associated with the removal of internal introns 2A, 2B and 3 are not limiting due to overexpression of *SMN* circRNAs. All three circRNAs of *SMN* we examined utilize 5′ss of exon 4 for backsplicing. A single G-to-C substitution at the invariant first position of the 5′ss of intron 4 completely suppressed backsplicing events. Interestingly, G-to-C substitution appeared to suppress skipping of exon 3 in the context of the linear transcripts generated in HeLa cells transiently transfected with vectors expressing L2A-2B-3-4 and L2B-3-4 (Supplementary Figure S2D).

We have previously reported circRNAs encompassing individual *SMN* exons 2A, 2B, 3 and 4, although they were generated at insignificant levels [25]. G-to-C substitution abrogating the 5′ss of exon 4 could, in principle, favor a circularization event involving individual exons 2A, 2B and 3 due to the steric advantage conferred by the protracted stem formed by inverted repeats present in the overexpressed precursor RNA. Yet, we did not detect circRNAs comprised of single exons. These results suggest that the structural constraints imposed by the RNA:RNA duplex, which pairs the 5′ss of exon 4 with the 3′ss of an upstream exon, are not conducive for the usage of the alternative (internal) 5′ss generating small circRNAs.

Our results of fractionation studies confirm the overwhelming cytosolic localization of *SMN* circRNAs. We have previously reported potential binding sites of several microRNAs within C2A-2B-3-4, C2B-3-4 and C3-4 [42]. Considering microRNAs suppress translation, sponging of microRNAs by *SMN* circRNAs localized in the cytoplasm is likely to impact translation of multiple mRNAs. Some of these microRNAs, including miR-130b-5p, miR-15b-3p and miR-6813-5p, are predicted to interact with the unique backsplice junction of C2A-2B-3-4. Hence, C2A-2B-3-4 has the potential to regulate the expression of microRNA targets with high specificity. Based on the analysis of CLIP data, several proteins, including AGO1, ASF/SF2, SRSF3, U2AF2 and YTHDC1, are predicted to interact with C2A-2B-3-4, C2B-3-4 and C3-4 [42]. Additional proteins, including FUS and TAF, have potential interaction sites within C2A-2B-3-4 and C2B-3-4 [42]. Sequestration of these proteins is likely to impact essential processes, including transcription, pre-mRNA splicing, translation and stress granule formation. Despite the absence of Alu elements, the mouse *Smn* gene expresses mC2A-2B-3-4 [25]. Of note, we used prefix ‘m’ to denote mouse circRNAs for clarity. Hence, functions associated with C2A-2B-3-4 are likely to be conserved between two distantly related organisms. Interestingly, C2B-3-4 and C3-4 were not detected in mice, suggesting their origins after Alu elements were inserted in primates [25]. Future studies will reveal if some of the human-specific functions of *SMN* genes are linked in part to *SMN* circRNAs. Now that we have generated stable cell lines expressing three abundant circRNAs of *SMN*, the stage is set to uncover novel *SMN* gene functions independent of the SMN protein.

## Data Availability

The data presented in this study are available in Supplementary Materials. Additionally, they can be found at the GenBank (https://www.ncbi.nlm.nih.gov, accessed on 20 November 2022) or directly requested from the corresponding author.

## Supplementary Materials

The following supporting information can be downloaded at: https://www.mdpi.com/article/10.3390/genes13071145/s1, Supplementary Figure S1: Construction of pC2B-3-4 vector to express C2B-3-4; Supplementary Figure S2: Relative expression of circular and linear transcripts from vectors carrying G1C mutation at the first position of intron 4; Supplementary Figure S3: Steps involved to establish stable cell lines to overexpress a gene of interest; Supplementary Figure S4: Characterization of linear transcripts in stable cell lines; Supplementary Figure S5: Identification of circRNAs from stable cell lines using primers targeting exons 2A and 2B; Supplementary Table S1: Abbreviations used in publication; Supplementary Table S2: List of overexpression vectors; Supplementary Table S3: List of primers; Supplementary Table S4: List of stable cell lines used.
