# Supplementary material for: Internal Introns Promote Backsplicing to Generate Circular RNAs from Spinal Muscular Atrophy Gene
Source: Genes (Basel). 2022 Jun 25;13(7):1145. doi: 10.3390/genes13071145 (PMC9323214; doi:10.3390/genes13071145)
Supplement: Supplementary file 1 [file genes-13-01145-s001.zip › Supplementary Figures 06-22-2022.pdf]

Supplementary Figures

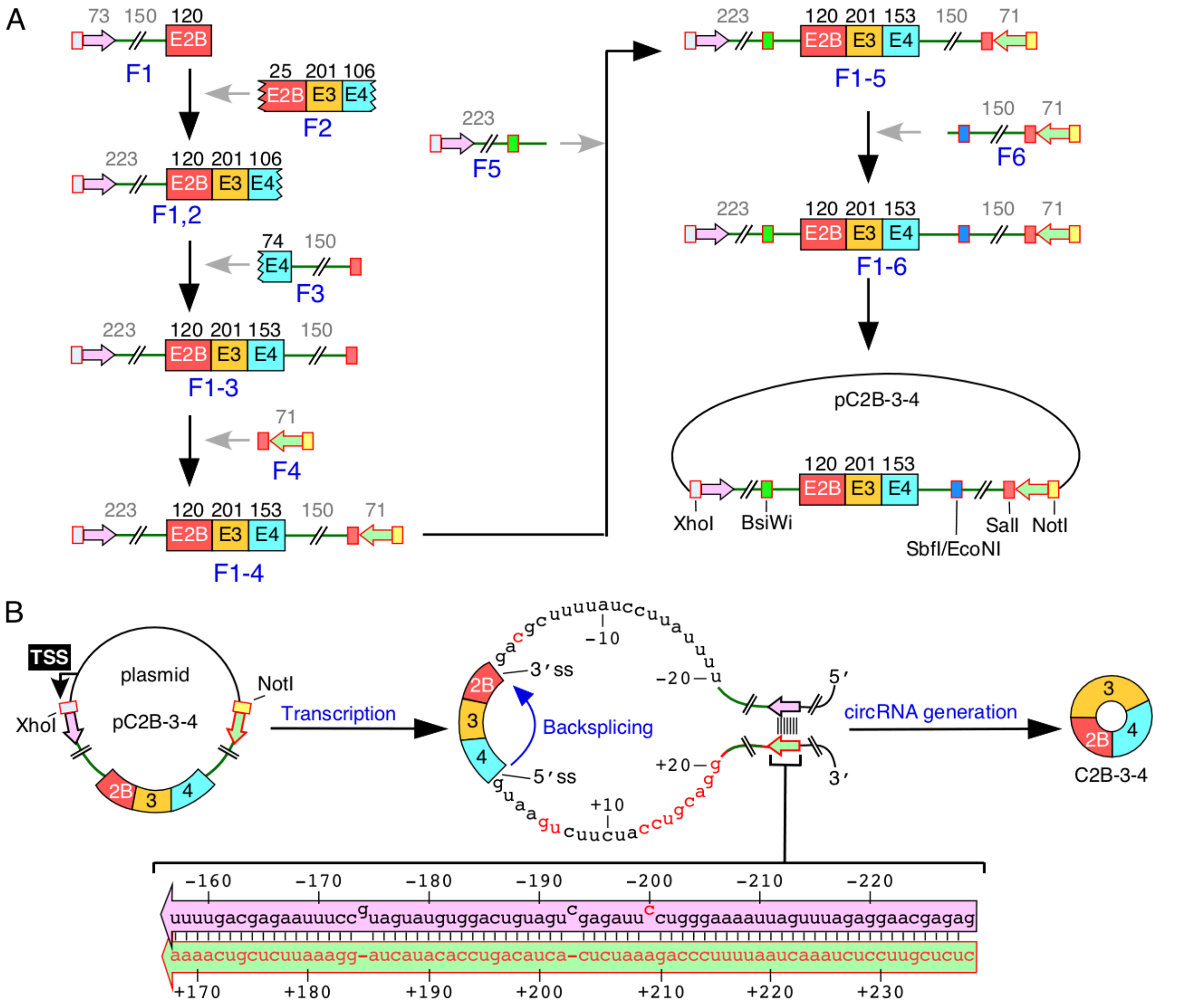

Supplementary Figure S1. Construction of pC2B-3-4 vector to express C2B-3-4. (**A**) Steps involved to generate C2B-3-4 vector. Fragments were generated by PCR and cloned into pCI backbone via Multiple Cloning Sites (MCSs). Exon-only and intron-containing fragments were generated using cDNA and genomic DNA as templates, respectively. A pair of complementary sequences are shown in colored arrows. Exons are depicted by colored boxes. Introns are shown by green lines. Sizes of exons and introns are given on the top. pCI backbone is shown in black. Restriction endonuclease sites are indicated in colored thin boxes. Abbreviation: F, Fragment. (**B**) Diagrammatic representation of transcription from pC2B-3-4 and generation of C2B-3-4. Left panel is the diagrammatic representation of pC2B-3-4 showing transcription start site. Middle panel shows splice sites and the base pairing of transcribed RNA. Nucleotides highlighted in red indicate mutations that strengthen the splice sites or create a cloning site. For clarity, the first position of 3′ss of exon 2B was set as position "-1", while the first position of exon 4 5′ss as position "+1". Right panel shows the generated circRNA C2B-3-4. Abbreviations: 5′ splice site (5′ss), 3′ splice site (3′ss).

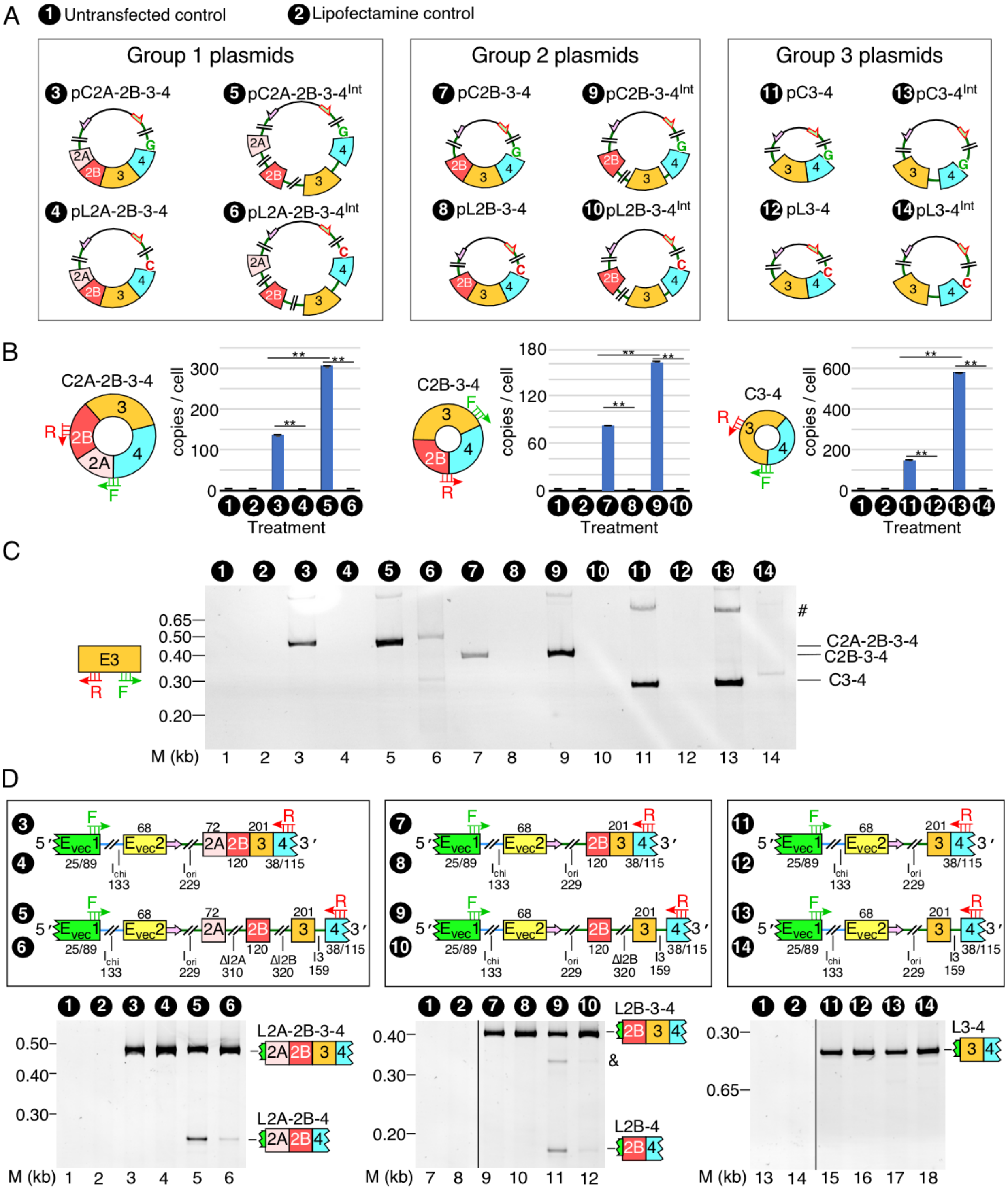

Supplementary Figure S2. Relative expression of circular and linear transcripts from vectors carrying G1C mutation at the first position of intron 4. (**A**) Diagrammatic representations of *SMN* circRNA expression vectors and their G1C mutation-bearing counterparts. These vectors are organized in three groups. Group 1 vectors contain four *SMN* exons (exons 2A, 2B, 3 and 4) and include pC2A-2B-3-4, pL2A-2B-3-4, pC2A-2B-3-4^{Int} and pL2A-2B-3-4^{Int} plasmids. Group 2 vectors contain three *SMN* exons (exons 2B, 3 and 4) and include pC2B-3-4, pL2B-3-4, pC2B-3-4^{Int} and pL2B-3-4^{Int}. Group 3 vectors contain two *SMN* exons (exons 3 and 4) and include pC3-4, pL3-4, pC3-4^{Int} and pL3-4^{Int}.

Vectors harboring *SMN* introns are depicted by superscript "^{Int}". "G" nucleotide shown in green signifies the functioning 5´ss of exon 4, while "C" nucleotide shown in red indicates G1C mutation that abrogates the 5´ss of exon 4. (**B**) Overexpression of *SMN* circRNAs. Each panel in (B) has a diagrammatic representation of the corresponding circRNA and a bar diagram showing the copy number of this circRNA per cell as determined by qPCR. Annealing positions of primers used are indicated. Error bars represent standard error of the mean. Statistical significance: **, $p<0.01$. Numbers at the bottom of each bar diagram is explained in panel (A). (**C**) Identification of circRNAs. A representative gel showing the results of semi-quantitative PCR using divergent primers targeting exon 3. Numbers on the top of the gel are same as in panel (A). Primers annealing sites are indicated on the left. The size marker (M) is indicated on the left side of gel, while the identity of the bands on the right side. The bands likely derived from duplicate of indicated circular transcript are marked by "#". (**D**) Detection of linear transcripts. Top panels show annealing positions of primers used for PCR to detect linear transcripts. Numbers are the same as in panel A. Bottom panels are representative gels showing the results of semi-quantitative PCR. The size marker (M) is indicated on the left side of gel, while the identity of the bands on the right side. An unknown band is marked as "&".

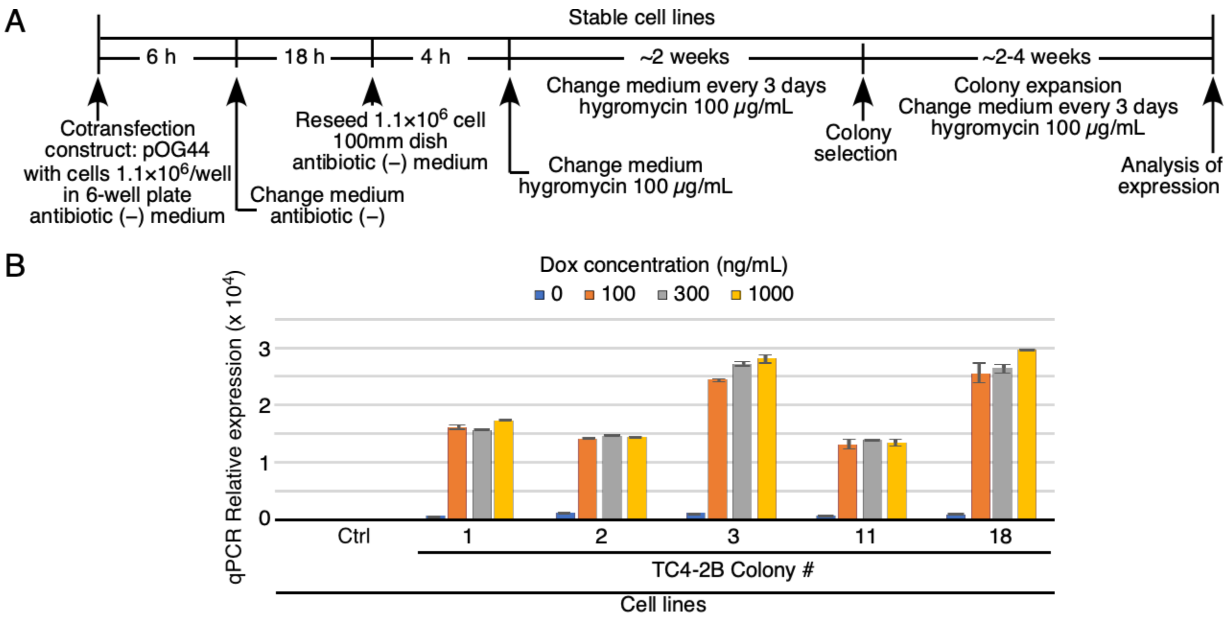

Supplementary Figure S3. Steps involved to establish stable cell lines to overexpress a gene of interest. (**A**) An overview of the timeline to generate cell lines to express GOI. (**B**) The dose effect of Dox on inducing the expression of C2B-3-4 in five TC4-2B colonies. Three concentrations were used, 0.1, 0.3 and 1 μg/mL. Untransfected T-REx cells were included as control (Ctrl). The expression levels of C2B-3-4 were quantified by Real-time PCR (qPCR) using $2^{-\Delta\Delta Ct}$ method, which were plotted by bar graph for Ctrl and different TC4-2B colonies. Abbreviation: GOI, gene of interest.

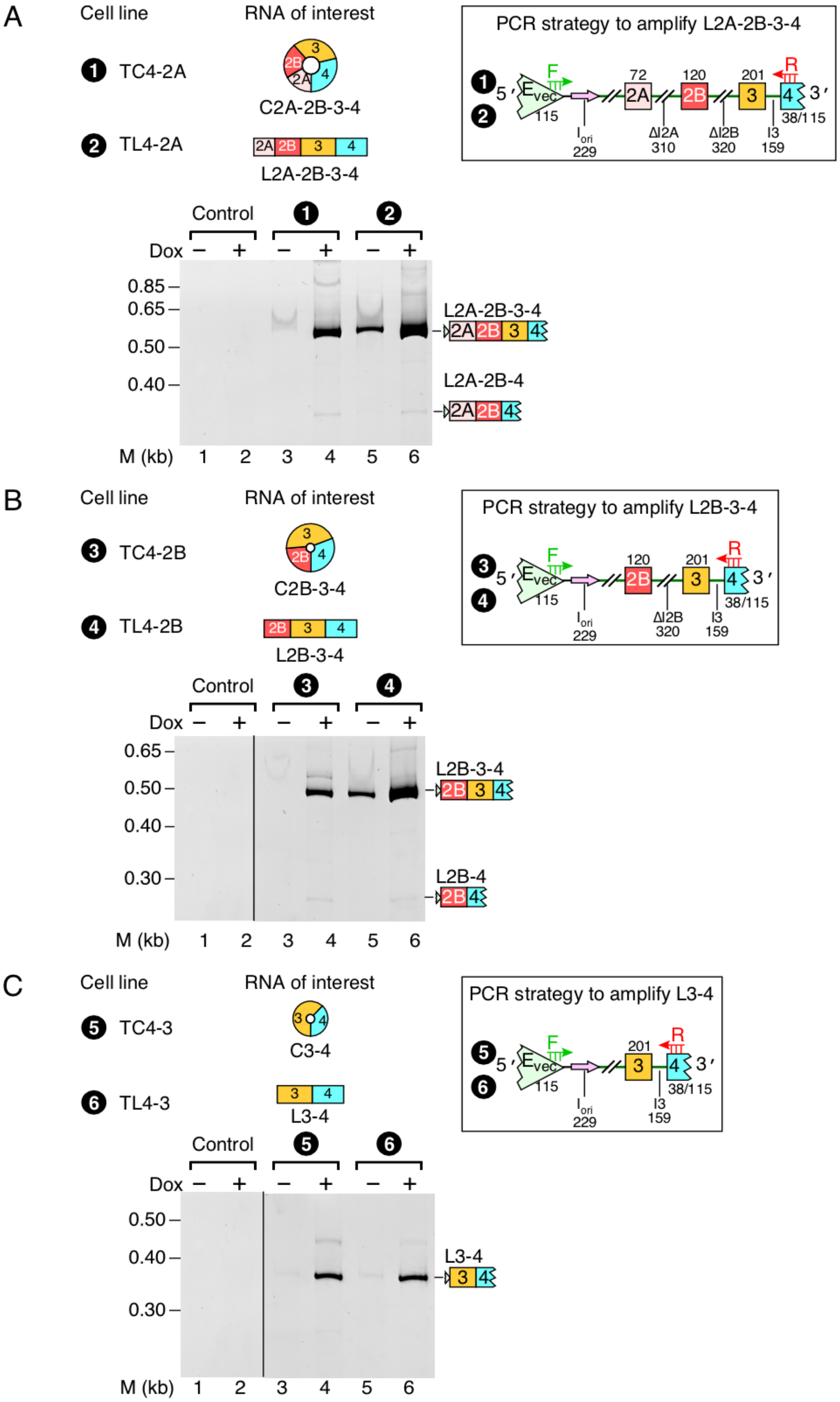

Supplementary Figure S4. Characterization of linear transcripts in stable cell lines. (**A**) Identification of linear transcripts generated in TC4-2A and TL4-2A cell lines. Top left panels show cell lines used and their respective transcripts overexpressed. Top right panel shows primer annealing positions to amplify linear transcripts. Bottom panel is a gel picture of semi-quantitative PCR showing linear transcripts. Treatments are labeled on the top of the gels. The size marker (M) is indicated on the left side of gel, while the identity of the bands on the right side. (**B**) Identification

of linear transcripts generated in TC4-2B and TL4-2B cell lines. (**C**) Identification of linear transcripts generated in TC4-3 and TL4-3 cell lines. Labeling is the same as in (A). Cell lines are organized in three groups. Group 1 cell lines produce circular or linear transcripts with four *SMN* exons (exons 2A, 2B, 3 and 4) and include TC4-2A and TL4-2A. Group 2 cell lines produce circular or linear transcripts with three *SMN* exons (exons 2B, 3 and 4) and include TC4-2B and TL4-2B. Group 3 cell lines produce circular or linear transcripts with two *SMN* exons (exons 3 and 4) and include TC4-3 and TL4-3.

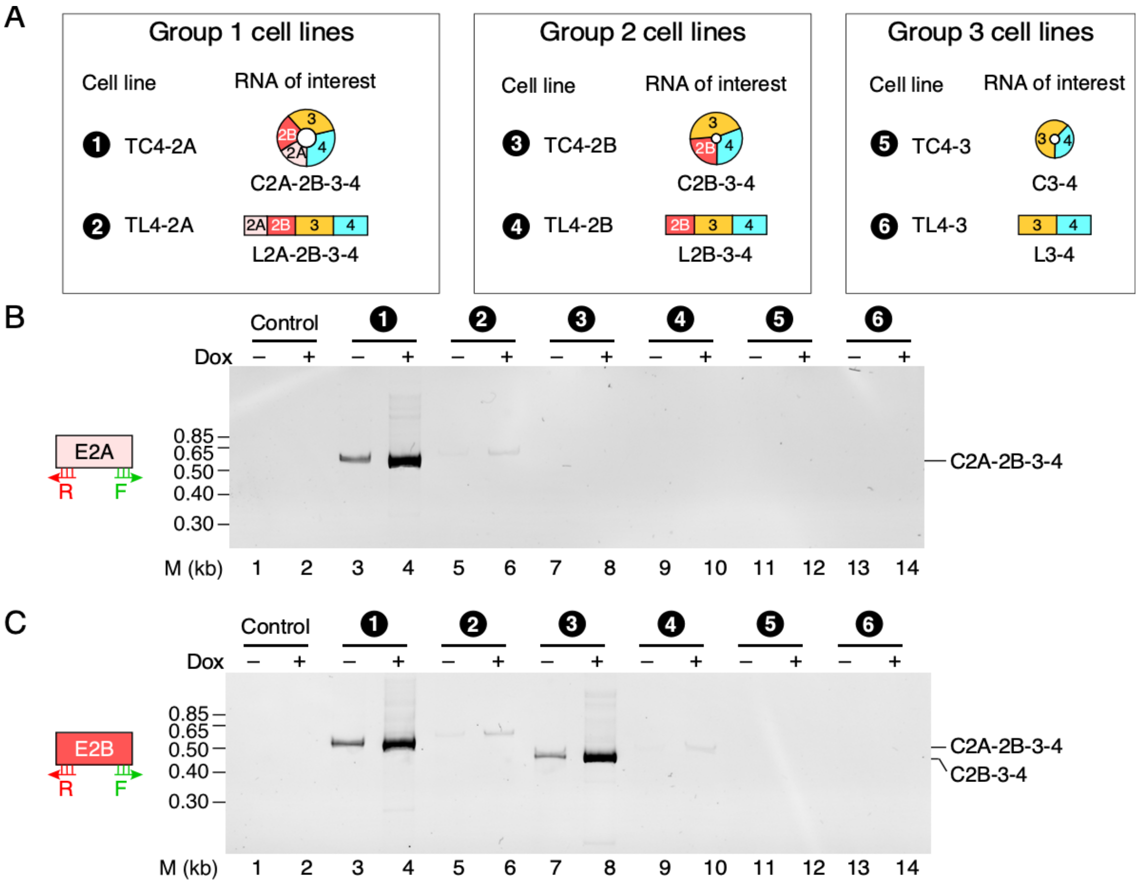

Supplementary Figure S5. Identification of circRNAs from stable cell lines using primers targeting exons 2A and 2B. (**A**) An overview of stable cell lines and RNA of interest. Cell lines are organized in three groups. Groups 1, 2 and 3 contains four, three and two exons of *SMN*, respectively. The annotation is the same as in Supplementary Figure S4. (**B**) Identification of circRNAs using divergent primers targeting exon 2A. Treatments are marked on the top of gel. The size marker (M) is indicated on the left side of gel, while the identity of the bands on the right side. Primer annealing sites are indicated at the left. (**C**) Identification of circRNAs using divergent primers targeting exon 2B. Labeling is the same as in (B).
