# Supplementary material for: Internal Introns Promote Backsplicing to Generate Circular RNAs from Spinal Muscular Atrophy Gene
Source: Genes (Basel). 2022 Jun 25;13(7):1145. doi: 10.3390/genes13071145 (PMC9323214; doi:10.3390/genes13071145)
Supplement: Supplementary file 1 [file genes-13-01145-s001.zip › Supplementary Tables 06-22-2022.pdf]

Supplementary Tables

Supplementary Table S1. Abbreviations used in publication.

Abbreviation	Full Name	Figures
3′ss	3′ splice site	
5′ss	5′ splice site	
Δ3	*SMN* linear transcript skipping of exon 3	2, 3 and 5
Δ3,7	*SMN* linear transcript skipping of exons 3 and 7	2, 3 and 5
Δ3,5	*SMN* linear transcript skipping of exons 3 and 5	2, 3 and 5
Δ3,5,7	*SMN* linear transcript skipping of exons 3, 5 and 7	2, 3 and 5
Δ5	*SMN* linear transcript skipping of exon 5	2, 3 and 5
Δ5,7	*SMN* linear transcript skipping of exons 5 and 7	2, 3 and 5
Δ7	*SMN* linear transcript skipping of exon 7	2, 3 and 5
ΔI2A	Truncated intron 2A	S2 and S4
ΔI2B	Truncated intron 2B	S2 and S4
ΔI4	Truncated intron 4	S2 and S4
bp	Base pair	
circRNA	Circular RNA	
C2A-2B-3-4	circRNA encompassing exons 2A, 2B, 3 and 4 of human *SMN* gene	
C2B-3-4	circRNA encompassing exons 2B, 3 and 4 of human *SMN* gene	
C3-4	circRNA encompassing exons 3 and 4 of human *SMN* gene	
CGFP	circRNA encompassing *eGFP* gene	2
CHIPK2	circRNA encompassing exon 2 of human *HIPK2* gene	2
Dox	Doxycycline	4-6, and S3-5
EJC	Exon-junction complex	
E_{vec}	Sequence transcribed between the TSS and I_{ori} at the integration site in the stable cell lines	S4
E_{vec1}	Sequence upstream of the chimeric intron from the pCI plasmid backbone	S2
E_{vec2}	Sequence downstream of the chimeric intron from the pCI plasmid backbone	S2
F	Forward primer	
F + number	DNA fragment in cloning strategy	S1
FL	Full length linear transcript of *SMN*	2, 3 and 5
GOI	Gene of interest	
G1C	G to C mutation at the first position of 5′ss	
h	Hour(s)	
H3	Histone H3 protein	6

I_{chi}	Chimeric intron from the pCI plasmid backbone	S2
I_{ori}	Sequence bearing the truncated intron 2A including an original sequence at 5′ end, which is reverse complementary to the artificial sequence inserted in intron 4	S2 and S4
L2A-2B-3-4	Linear RNA encompassing exons 2A, 2B, 3 and 4 of human *SMN* gene	S2 and S4
L2B-3-4	Linear RNA encompassing exons 2B, 3 and 4 of human *SMN* gene	S2 and S4
L3-4	Linear RNA encompassing exons 3 and 4 of human *SMN* gene	S2 and S4
MESDA	Multi-exon skipping detection assay	
min	Minute(s)	
nt	Nucleotide	
Pol II	RNA Polymerase II	
R	Reverse prime	
SMA	Spinal Muscular Atrophy	
SMN (Italics)	Survival motor neuron gene or transcript	
SMN	Survival motor neuron protein	
TC4-2A	Stable cell lines expressing C2A-2B-3-4	5, S4 and S5
TC4-2B	Stable cell lines expressing C2B-3-4	5, S4 and S5
TC4-3	Stable cell lines expressing C3-4	5, S4 and S5
TL4-2A	Stable cell lines expressing L2A-2B-3-4	5, S4 and S5
TL4-2B	Stable cell lines expressing L2B-3-4	5, S4 and S5
TL4-3	Stable cell lines expressing L3-4	5, S4 and S5
T-REx	T-REx™-293 cell line	
TSS	Transcription start site	2, S1 and S2

Supplementary Table S2. List of overexpression vectors.

Plasmid	RNA of interest	Origin of inserted fragments	Backbone	Exons	G1C	Accession Number
pCGFP	CGFP	pEGFP-C1	pCI	*eGFP* gene 81-555 bp	N	ON526832
pCHIPK2	CHIPK2	*HIPK2*	pCI	exon 2	N	ON526833
pC2A-2B-3-4	C2A-2B-3-4	*SMN*	pCI	exons 2A, 2B, 3 and 4	N	ON526814
pL2A-2B-3-4	L2A-2B-3-4	*SMN*	pCI	exons 2A, 2B, 3 and 4	Y	ON526815
pC2A-2B-3-4^{Int}	C2A-2B-3-4	*SMN*	pCI	exons 2A, 2B, 3 and 4	N	ON526816
pL2A-2B-3-4^{Int}	L2A-2B-3-4	*SMN*	pCI	exons 2A, 2B, 3 and 4	Y	ON526817
pC2B-3-4	CB-3-4	*SMN*	pCI	exons 2B, 3 and 4	N	ON526818
pL2B-3-4	L2B-3-4	*SMN*	pCI	exons 2B, 3 and 4	Y	ON526819
pC2B-3-4^{Int}	CB-3-4	*SMN*	pCI	exons 2B, 3 and 4	N	ON526820
pL2B-3-4^{Int}	L2B-3-4	*SMN*	pCI	exons 2B, 3 and 4	Y	ON526821
pC3-4	C3-4	*SMN*	pCI	exons 3 and 4	N	ON526822
pL3-4	L3-4	*SMN*	pCI	exons 3 and 4	Y	ON526823
pC3-4^{Int}	C3-4	*SMN*	pCI	exons 3 and 4	N	ON526824
pL3-4^{Int}	L3-4	*SMN*	pCI	exons 3 and 4	Y	ON526825
pTC2A-2B-3-4^{Int}	C2A-B-3-4	*SMN*	pcDNA5/FRT/TO	exons 2A, 2B, 3 and 4	N	ON526826
pTL2A-2B-3-4 ^{Int}	L2A-2B-3-4	*SMN*	pcDNA5/FRT/TO	exons 2A, 2B, 3 and 4	Y	ON526827
pTC2B-3-4 ^{Int}	C2B-3-4	*SMN*	pcDNA5/FRT/TO	exons 2B, 3 and 4	N	ON526828
pTL2B-3-4 ^{Int}	L2B-3-4	*SMN*	pcDNA5/FRT/TO	exons 2B, 3 and 4	Y	ON526829
pTC3-4 ^{Int}	C3-4	*SMN*	pcDNA5/FRT/TO	exons 3 and 4	N	ON526830
pTL3-4 ^{Int}	L3-4	*SMN*	pcDNA5/FRT/TO	exons 3 and 4	Y	ON526831

Abbreviations: Int, intercalating introns; G1C, a G-to-C mutation at the first intronic position of 5′ss of exon 4.

Supplementary Table S3. List of primers.

#	primer name	Target sequence	Usage	Primer sequence (5′ to 3′)
1	E1-F2	*SMN*, exon 1	PCR	CGGGTTTGCTATGGCGATG
2	5 E2A-End	*SMN*, exon 2A	PCR	TATGATAAAGCTGTGGCTTCAT
3	3 SMN2 Exon2a	*SMN*, exon 2A	PCR	CAGTGCTGTATCATCCCAAATGTCAG
4	5 E2B-End	*SMN*, exon 2B	PCR	GAATACTGCAGCTTCCTTACAAC
5	3 E2B-Start	SMN, exon 2B	PCR	CAAATGTCACCATTCTTTAGAGC
6	5 E3-End	*SMN*, exon 3	PCR	CTTTCCCCAATCTGTGAAGTAGCTA
7	3 E3-Start	*SMN*, exon 3	PCR	CAACCGTCTTCTGACCAAATGG
8	5 Ex4	*SMN*, exon 4	PCR	GGCCAAGACTGGGACCAGG
9	3 exon4SMN	*SMN*, exon 4	PCR	TCACTTTCATCTGTTGAAACTTGG
10	5 LNCX	pcDNA5/FRT/TO	PCR	GCTCGTTTAGTGAACCGTCAGATC
11	3 Ex8-25	*SMN*, exon 8	PCR	TAGTGCTGCTCTATGCCAGCATTTC
12	5 eGFP_end	pEGFP-C1	PCR	GATCCGCCACAACATCGAGG
13	3 eGFP_start	pEGFP-C1	PCR	TCAGGGTCAGCTTGCCGTAG
14	5 HIPK2-E2-end	*HIPK2*, exon 2	PCR	ACCATACAGAGTCAAGGTC
15	3 HIPK2-E2_start	*HIPK2*, exon 2	PCR	CTTTGCTGTGGGAGCCGTAC
16	3 SMN2 Int 4	*SMN*, intron 4	qPCR	GCTACAAAAGTTTCATGGGAGAGCTAC
17	5 SMN2 Exon 4	*SMN*, exon 4	qPCR	CCAAATCTGCTCCATGGAACTCTTTTC
18	5 E4+2A	*SMN*, exons 2A and 4	qPCR	CTGGGACCAGGAAAGAGCG
19	3 E2B (end)	*SMN*, exon 2B	qPCR	CTGTTGTAAGGAAGCTGCAGTATTC
20	5 E4+2B	*SMN*, exons 2B and 4	qPCR	CTGGGACCAGGAAAGCATG
21	5 E4+3	*SMN*, exons 3 and 4	qPCR	CTGGGACCAGGAAAGTGGA
22	3 E3 (middle)	*SMN*, exon 3	qPCR	GGACAGATTTTGCTCCTCTCTATTTC
23	hGAPDH-1	*GAPDH*, exon 8	qPCR	AACAGCGACACCCACTCCTC
24	hGAPDH-2	*GAPDH*, exons 8 and 9	qPCR	CATACCAGGAAATGAGCTTGACAA
25	5 pCI-intr-Up	pCI	qPCR	AAGTTGGTCGTGAGGCACTG
26	3 E2A-RT	*SMN*, exon 2A	RT	TGCTTTTATCAGTGCTGTATCAT
27	3 E2B-RT	*SMN*, exon 2B	RT	GGCTTTTATTCTTCTTAGCAGG

28	3 E3-RT	*SMN*, exon 3	RT	TCCATATCCAGTGTAAACCACA AC
29	3 ex8_541(DdeI)	*SMN*, exon 8	RT	CTACAACACCCTTCTCACAGCTC
30	3 eGFP_RT	pEGFP-C1	RT	AGAAGTCGTGCTGCTTCATG
31	3 HIPK2-E3_RT	*HIPK2*, exon 2	RT	ATGTTCTTGCTCTGGCTATACAC
32	5 Str3ss_BsiWI	*SMN*, intron 2A	Cloning	ATGCCGTACGTTTTTATTCCTATT TTCGCAG
33	3 Str3ss_BsiWI	*SMN*, intron 2A	Cloning	CTGCGAAAATAGGAATAAAAAC GTACGTGTACATGTTACAGG
34	5 Str5ss_SbfI+EcoNI	*SMN*, intron 4	Cloning	CTTCTACCTGCAGGAGGTGAAA GTTTTCCAGAAAATAG
35	3 Str5ss_SbfI+EcoNI	*SMN*, intron 4	Cloning	TCACCTCCTGCAGGTAGAAGAC TTACCTTTCC
36	3 pN2 5′ss-G1C	*SMN*, intron 4	Cloning	GCATCCTCCTGCAGGTAGAAGA CTTAGCTTTCCTGG
37	3 Exon4_G1C	*SMN*, exon 4	Cloning	ACCTCCTGCAGGTAGAAGACTT AG
38	3 I4Str5′ss	*SMN*, intron 4	Cloning	CACCTCCTGCAGGTAGAAGAC
39	pCI-UP	pCI	Cloning	TGACATCCACTTTGCCTTTCTCTC
40	pCI-DN	pCI	Cloning	AGCATCACAAATTTCACAAATA AA
41	5 I2A (26)-XhoI	*SMN*, intron 2A	Cloning	ATGCCTCGAGGAGAGCAAGGAG ATTTGATTAAAAGG
42	3 5ss _SbfI+EcoNI	*SMN*, intron 4	Cloning	TCACCTCCTGCAGGTAGAAGAC TTACCTTTCC
43	5 I 4_SbfIEcoNI	*SMN*, intron 4	Cloning	CTTCTACCTGCAGGAGGTGAAA GTTTTCC
44	5 eGFP_BsiWI	pEGFP-C1	Cloning	TTCCTATTTTCGCAGAAACGGCC ACAAGTTC
45	3 eGFP_SbfI+EcoNI	pEGFP-C1	Cloning	AGGTAGAAGACTTACTCGGCGA GCTGC
46	5 HIPK2_BsiWI	*HIPK2*, exon 2	Cloning	TTCCTATTTTCGCAGGTATGGCC TCACATG
47	3 HIPK2_SbfI+EcoNI	*HIPK2*, exon 2	Cloning	AGGTAGAAGACTTACCTGTAAT ATCTGGACTGC
48	5 I2A(-3C)-2A_N2	*SMN*, intron 2A	Cloning	TTCCTATTTTCGCAGAGCGATGA TTCTGAC
49	5 SMN2 Exon 4	*SMN*, exon 4	Cloning	CCAAATCTGCTCCATGGAACTCT TTTC
50	3 SMN2 Exon 4	*SMN*, exon 4	Cloning	GAAAAGAGTTCCATGGAGCAGA TTTGG
51	5comple-SalI	Constructs	Cloning	ATGCGTCGACAAAACTGCTCTTA AAGGCATCATACACCTGACATC AGCTCTAAAGACCCT

52	3comple-NotI	Constructs	Cloning	ATGCGCGGCCGCGAGAGCAAGG AGATTTGATTAAAAGGGTCTTTA GAGCTGATGTCAGG

Supplementary Table S4. List of stable cell lines used.

Cell line	RNA of interest	circRNA expression Dox (+) (copies/cell)	Origin	Exons	Intercalating introns	G1C mutation at 5′ss of exon 4
T-REx™-293	endogenous	0.01-0.03	N/A	N/A	N/A	N/A
TC4-2A	C2A-2B-3-4	1139.36	*SMN*	exons 2A, 2B, 3 and 4	Y	N
TL4-2A	L2A-2B-3-4	0.04	*SMN*	exons 2A, 2B, 3 and 4	Y	Y
TC4-2B	C2B-3-4	941.44	*SMN*	exons 2B, 3 and 4	Y	N
TL4-2B	L2B-3-4	0.1	*SMN*	exons 2B, 3 and 4	Y	Y
TC4-3	C3-4	520.49	*SMN*	exons 3 and 4	Y	N
TL4-3	L3-4	0.01	*SMN*	exons 3 and 4	Y	Y

The host cell line was T-REx™-293. Backbone for all incorporated plasmids was pcDNA5/FRT/TO. The induction reagent for all stable cell lines was doxycycline.
